# Main factors predicting somatic, psychological, and cognitive patient outcomes after significant injury: a pilot study of a simple prognostic tool

**DOI:** 10.1093/bjsopen/zrab109

**Published:** 2021-12-03

**Authors:** Thomas Gross, Felix Amsler

**Affiliations:** Trauma Unit, Cantonal Hospital Aarau, Aarau and University of Basel, Basel, Switzerland; Amsler Consulting, Basel, Switzerland

## Abstract

**Background:**

There are still insufficient data on the complexity and predictability of patient-related outcomes following trauma. The aim of this study was to assess longer-term outcomes in patients with significant injury and to develop a simple scoring method to identify patients at high risk of subsequent deficits 1–2 years after injury.

**Methods:**

We conducted a prospective cohort study of survivors of significant injury (New Injury Severity Score, NISS greater than or equal to 8), with analysis of patients’ 1- to 2-year health-related quality of life (HRQoL) and their functional outcomes based on Short Form-36 (SF-36), Trauma Outcome Profile (TOP), and Quality Of Life after Brain Injury (QOLIBRI). Documented variables suspected or known from the literature to be possible factors associated with outcome were first analysed by univariate analysis, and significant variables were entered into a stepwise logistic regression analysis. Scores predicting longer-term impaired outcome were constructed from risk factors resulting from multivariate analysis.

**Results:**

Depending on the patient-reported outcome measure (PROM) used, up to 30 per cent of 1052 study patients (mean NISS 18.6) indicated somatic, 27 per cent psychological, and 54 per cent cognitive deficits. The investigated sociodemographic, injury-related, treatment, and early hospital outcome variables demonstrated only low associations with longer-term outcome in univariate analysis that were highest for preinjury pain or function (*R* = 0.4) and outcome at hospital discharge (*R* = 0.3). After logistic regression, the study variables explained a maximum variance of 23 per cent for somatic, 11 per cent for psychological, and 14 per cent for cognitive longer-term outcomes. The resulting Aarau trauma prognostic longer-term outcome scoring (ATPLOS) system, developed by checking eight risk factors, had a specificity of up to 80 per cent, and importantly may facilitate early detection of patients at risk of a poorer longer-term outcome.

**Conclusion:**

Despite the high rate of deficits recorded for survivors of significant injury, particularly in loss of cognitive function, the multiple variables analysed only led to a limited characterization of patient-related longer-term outcomes. Until more is known about additional individual influencing factors, the proposed scoring system may serve well for clinical evaluation.

**Registration number:**

NCT 02165137 (http://www.clinicaltrials.gov)

## Teaser

This study of survivors of significant injury showed a high rate of somatic, psychological, and especially cognitive deficits. Early sociodemographic, trauma, and treatment variables correlated only to a limited extent with outcome 1–2 years after trauma. However, multivariate analysis allowed the development of a potential diagnostic instrument, the Aarau trauma prognostic longer-term outcome scoring (ATPLOS), to predict the longer-term outcome of patients using the patient-reported outcome measures (PROMs) Short Form-36 (SF-36) and Trauma Outcome Profile (TOP) with reasonable sensitivity, specificity, and likelihood ratio. Application of such predictive factors may therefore help attending clinicians to initiate well targeted therapeutic interventions earlier and more effectively.

## Introduction

Traumatic injury is a leading cause of disability, and survivors of trauma may experience significant restrictions to their functional outcome and health-related quality of life (HRQoL)^1^. Increasing evidence demonstrates a high prevalence of longer-term challenges related to patients’ daily activities such as persistent pain, diminished resilience, and anxiety and depression following injury^2–5^, but only a few trauma registries routinely collect postdischarge data such as patient-reported outcome measures (PROMs) and HRQoL. The largest repository of longitudinal follow-up results is the Victorian State Trauma Registry^2^^,^^6^^,^^7^ based on standardized telephone interviews. The United States very recently launched national initiatives in this regard^8^^,^^9^, modelled on the Boston trauma centre experience^10^^,^^11^. Extensive European trauma registries, such as the Trauma Audit & Research Network (TARN; https://www.tarn.ac.uk/Content.aspx?ca=4) and the German Society for Trauma Surgery Registry^12^, have started to include longer-term follow-up data of patients. These registries all take a different approach, that is there are no standardized inclusion criteria or measures of disability or health outcome^9^^,^^13^. Given the absence of an internationally agreed framework for the assessment of disability and function^14^, and despite the World Health Organization’s initiative for an integrative biopsychosocial model of functioning, disability, and health (The International Classification of Functioning, Disability and Health, ICF; https://ec.europa.eu/cefdigital/wiki/display/EHSEMANTIC/ICF+-+the+WHO+International+Classification+of+Functioning%2C+Disability+and+Health), it is only very recently that possible categories for an agreed ICF-based minimum data set were presented^15^. Validated tools to support the selection and implementation of various PROMs^16^^,^^17^ are still under development^18^. Therefore, on the one hand, PROMs are still underutilised in trauma populations^1^, but, on the other hand, numerous PROMs have been used in trauma cohorts despite a lack of agreement. In terms of clinical practice, consensus conferences and reviews on the topic have recommended a combination of generic and condition-specific tools^1^^,^^9^^,^^19^.

Surprisingly, despite the important role of cognitive function in daily life, there is no large trauma centre evaluation that has specifically investigated cognitive impairment after injury such as limitations in executive function, memory, new learning, concentration, or making decisions that affect everyday life^9^. At most, possible longer-term cognitive effects have been investigated indirectly by psychological subscorings. It is recognized that cognitive deficits are found not only following traumatic brain injury (TBI), but also in non-TBI patients^20–22^.

The aim of the present study was to assess longer-term outcomes in patients with significant injury (New Injury Severity Score (NISS) of greater than or equal to 8), using both generic and disease-specific PROMs^5^^,^^20–24^. Specifically, we investigated the proportion of patients reporting somatic, psychological, and cognitive deficits, how these longer-term consequences might be predicted during the initial postinjury phase, and whether a simple scoring method can be used early to identify those patients at high risk of subsequent deficits 1–2 years after injury.

## Methods

This study was conducted at a dedicated trauma centre and approved by the regional ethics committee (Ethics Commission Northwest and Central Switzerland, PB_2018–00079). The teaching hospital serves a catchment population of about 750 000 inhabitants. From 1 January 2010 to 30 September 2018, all significantly injured patients (NISS greater than or equal to 8) over 15 years of age and admitted to the emergency department within 24 hours of injury were recruited to this prospective quality control study and consecutively evaluated. Hospital procedural guidelines followed international standards^23^^,^^24^.

All patients gave their informed consent to their inclusion in the study. The study was approved by the regional ethics committee and performed in accordance with the ethical standards laid down in the Declaration of Helsinki.

### Data management

Specially trained and experienced study nurses who were not involved in the treatment of patients were responsible for data management and trauma scores. Injury severity was determined based on maximum information available at the end of hospital stay. Demographic characteristics included age at time of injury (years) and sex (men/women). Co-morbidity was measured with the age-unadjusted Charlson Comorbidity Index^25^. Injury-related variables were: Glasgow Coma Scale (GCS)^26^ (using the first available score following injury), Abbreviated Injury Scale (AIS)^27^, Injury Severity Score (ISS)^28^, NISS^29^ (using the 2005 version and 2008 update of the trauma registry of the German Trauma Society), and Revised Injury Severity Classification (RISC)^30^. Trauma mechanism was graded as low (fall from less than 3 m, stab, blow, other) *versus* high (traffic, fall from greater than 3 m, gunshot). Multiple injury was defined as an AIS greater than 1 in at least two AIS body regions. Exclusion criteria for this study were patients who died up to the time of follow-up or those presenting with a Glasgow Outcome Score (GOS)^31^ of 2 (persistent vegetative state) when leaving the hospital. The survival status of non-responders at the time of follow-up was obtained from registries of public authorities and by contacting the next of kin or family practitioners.

Patient longer-term outcomes were assessed at 1 and/or 2 years following trauma by postal survey, complemented by phone interviews undertaken by specially trained study nurses for missing or non-plausible answers. Since follow-up examinations did not start until 2012, only 2-year follow-up data could be collected for patients who had sustained an injury in 2010. For all other patients, 1-year or, if missing, 2-year follow-up data were used. This was considered acceptable because earlier investigations had shown only minimal outcome differences between the first and second year after injury^32^. Standardized self-report questionnaires comprised a combination of validated generic and condition-specific, that is trauma-specific, PROMs. These consisted of a general HRQoL instrument, namely the Short Form-36 (SF-36)^33^ and the Trauma Outcome Profile (TOP)^21^^,^^34^, as well as the Quality Of Life after Brain Injury (QOLIBRI) score^35^ as a measurement of functional outcome following injury. All were used according to their cited original version.

The thresholds for impaired values for SF-36 were taken from the definitions of Pirente *et al*.^34^^,^^36^. TOP somatic was defined as impaired if at least two of the four dimensions of pain, function, activities, or body image were impaired. TOP psychological was defined as impaired if at least four of the six dimensions of depression, anxiousness, post-traumatic stress disorder (PTSD), social aspects, satisfaction, or cognitive function were impaired. Cognitive function includes tiredness, concentration problems, forgetfulness, and change of character, and was also used alone to describe cognitive impairment in addition to the cognitive dimension of the QOLIBRI. This measure includes the ability to: concentrate; express oneself and understand others; remember everyday things; make plans and find solutions to practical problems; make decisions; find one’s way around; and think at an appropriate speed.

### Statistics

Data are displayed as mean and s.d. for numeric variables; numbers and percentages are given for nominal variables. All statistical tests are two-tailed, and *P* < 0.050 was considered significant. For univariate statistics, missing cases were excluded variable-wise. For multivariate regression analysis, which used only dichotomized variables, missing values were replaced by the more frequent value of the whole cohort. Respondents were compared to non-respondents, with the paired *t* test for parametric data and with chi-square tests for binary values. *R*^2^ (explained variance) is shown to highlight the power of the results. Documented binary or dichotomized variables suspected or known from the literature to be possible factors associated with outcome were first analysed by univariate analysis using tetrachoric correlations, which can be interpreted analogously to Pearson correlations. Significant variables were entered into block and stepwise logistic regression analysis, with the entry criterion of *P* < 0.05 – first, including demographic and injury-related variables, and second, including treatment and hospital outcome-related variables as predictors of long-term somatically, psychologically, or cognitively impaired outcomes. Scores predicting longer-term impaired outcome were then constructed by giving 1 point for each significant variable as a risk factor in the multivariate analysis. The resultant ‘Aarau trauma prognostic longer-term outcome score’ (ATPLOS) is calculated by adding 1 point for each risk factor present (see results). The threshold values for the binary prediction score were defined according to the criterion that the number of risk factors had a higher percentage of impaired patients than the total sample. Sensitivity, specificity, likelihood ratios, and factors between low- and high-risk patients for longer-term impairment were calculated based on these binary prediction scores. Data were analysed using SPSS^®^ for Windows 26.0 (IBM, Armonk, New York, USA).

## Results

**Fig. 1:**
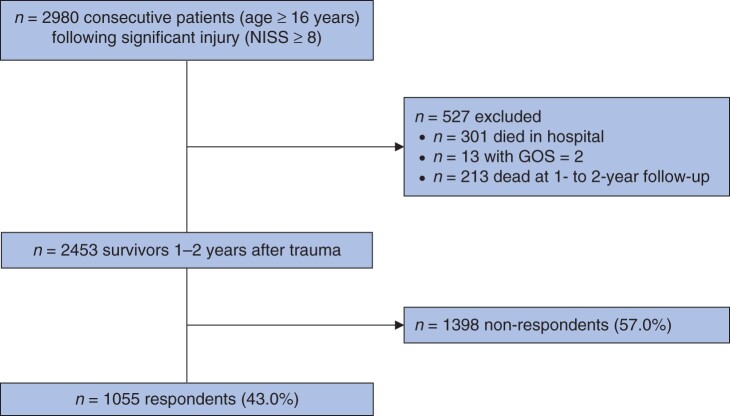
Patient flow chart NISS, New Injury Severity Score; GOS, Glasgow Outcome Scale.

Of 2453 eligible patients, a total of 1055 significantly injured individuals replied at longer-term follow-up (response rate 43.0 per cent; *Fig. 1*). Only 2-year follow-up data could be collected for 115 patients who had sustained an injury in 2010. For all other patients, 1-year (819 patients) or, if missing, 2-year (121 patients) follow-up data were used. Overall, respondents (mean age 54.1 ± 19.2 years; 66.3 per cent of men) differed only slightly from non-respondents (maximum *R*^2^ = 0.02; *Table S1*). Details of specific outcome measures and the criteria for, and percentages of, impaired outcomes used for the study cohort are listed in *Table 1*. Depending on single definitions, 28.3 to 29.6 per cent of patients showed somatic, 25.5 to 27.1 per cent psychological, and 49.4 to 53.9 per cent cognitive impairment.

**Table 1:** Description of outcomes for the study cohort of 1055 patients and variables used: mean values of patient-related longer-term outcome dimensions, criterion for impaired values, and proportion of patients with impaired values

Instrument	Measure	Mean (s.d.)	Criterion for impairment	Proportion of patients impaired, *n* (%)
**SF-36**	Somatic dimension*	46.0 (10.3)	< 40†	312 (29.6%)
	Psychological dimension*	47.0 (13.0)	< 40†	286 (27.1%)
**TOP**	Pain before injury	90.7 (15.7)	< 80	145 (13.7%)
	Function restriction before injury	93.0 (14.4)	< 80	101 (9.6%)
	Pain	82.3 (21.0)	< 80	300 (28.4%)
	Function	83.8 (21.2)	< 80	278 (26.4%)
	Activities	86.2 (21.0)	< 80	271 (25.7%)
	Body image	90.0 (22.7)	< 80	199 (18.9%)
	Number of dimensions of somatic impairment*	0.99 (1.22)	> 1†	298 (28.3%)
	Depression	82.4 (22.4)	< 80	335 (31.8%)
	Anxiousness	84.9 (20.1)	< 80	301 (28.5%)
	PTSD	78.7 (23.2)	< 80	416 (39.4%)
	Social aspects	79.3 (25.6)	< 80	384 (36.4%)
	Satisfaction	87.0 (26.2)	< 80	149 (14.1%)
	Cognitive function* ‡	69.8 (26.9)	< 80†	569 (53.9%)
	Number of dimensions of psychological impairment*	2.04 (1.94)	> 3†	269 (25.5%)
**QOLIBRI**	Q Cognition Ty*	75.6 (23.1)	< 80†	521 (49.4%)

*Central outcome dimensions.

†Threshold value for impaired outcome.

‡Also used separately in this investigation for cognitive outcome; SD, Standard deviation, SF-36, The Short Form-36; TOP, Trauma Outcome Profile; QOLIBRI, the Quality Of Life after Brain Injury score; PTSD, Post-Traumatic Stress Disorder.

Univariate analysis of the influence of categorical patient, injury, and treatment characteristics on longer-term outcome (*Table 2*) showed the highest correlations for pain or functional impairment prior to injury (maximum *R* = 0.36). Long hospital stay and/or no direct home discharge correlated significantly with all outcome dimensions (maximum *R* = 0.30). In terms of somatic impairment, the variable ‘musculoskeletal injury’ (AIS 5) had the next best correlation (maximum *R* = 0.21), whereas psychological impairment could only be predicted very weakly by the injury (AIS 5; maximum *R* = 0.08). In the case of cognitive impairment, it was ‘head and neck injury’ had a positive correlation? (AIS 1; maximum *R* = 0.14). On the other hand, ‘head and neck injury’ (AIS 1) correlated slightly negatively with somatic impairment (*R* = 0.07).

**Table 2:** Univariate correlation of dichotomized sociodemographic, injury, treatment, and hospital outcome variables with longer-term somatically, psychologically, and cognitively impaired outcomes (*n* = 1055)

	TOP: pain impairment	TOP: functional restriction	TOP: function or pain impairment	SF-36: somatic impairment	TOP: somatic impairment	SF-36: psychological impairment	TOP: psychological impairment	QOLIBRI: cognition impairment	TOP: cognitive impairment
**Age at time of trauma of at least 80 years§**	0.04	0.08†	0.07*	0.15‡	0.11‡	0.00	0.00	0.08†	0.15‡
**Sex (women)**	0.01	0.05	0.04	−0.01	0.04	0.07*	0.03	−0.02	0.08*
**Living in a partnership**	0.01	0.01	0.01	−0.01	0.00	−0.02	−0.03	−0.02	0.06
**No vocational education**	0.13‡	0.07*	0.10‡	0.05	0.11‡	0.13‡	0.14‡	0.11‡	0.10‡
**Age-unadjusted Charlson Comorbidity Index > 1**	0.07*	0.09†	0.10‡	0.14‡	0.09†	0.05	0.02	0.05	0.08*
**TOP: preinjury function or pain impairment before trauma**	0.35‡	0.36‡	0.35‡	0.22‡	0.35‡	0.17‡	0.20‡	0.18‡	0.21‡
**High trauma energy**	0.00	−0.03	0.00	0.03	−0.03	−0.03	−0.02	−0.04	−0.05
**Traffic accident, except car**	−0.04	−0.03	−0.02	0.01	−0.05	0.00	−0.01	−0.01	−0.04
**ISS ≥ 16**	−0.04	0.02	0.00	0.02	0.01	0.02	0.06	0.03	0.10‡
**ICU stay**	−0.03	0.04	0.01	0.01	0.02	0.09†	0.10‡	0.07*	0.14‡
**Intubation**	−0.01	0.05	0.03	0.06*	0.06	0.04	0.07*	0.04	0.09†
**1st GCS < 13**	−0.05	0.01	−0.02	0.01	0.01	0.01	0.03	0.05	0.11‡
**AIS 1 head/neck > 1**	−0.10‡	−0.08†	−0.10‡	−0.14‡	−0.08*	0.02	0.05	0.06*	0.12‡
**AIS 1 head/neck > 2**	−0.11‡	−0.08*	−0.09†	−0.11‡	−0.08†	0.04	0.07*	0.12‡	0.14‡
**AIS 3 chest > 1**	0.02	0.04	0.02	0.04	0.01	−0.05	−0.01	−0.03	−0.03
**AIS 3 chest > 2**	−0.02	0.02	−0.01	0.02	−0.01	−0.05	−0.02	−0.03	−0.04
**AIS 4 abdomen > 1**	0.06*	0.05	0.08*	0.04	0.04	−0.02	0.00	−0.06	−0.01
**AIS 4 abdomen > 2**	0.00	−0.03	0.01	0.00	−0.02	−0.03	−0.02	−0.07*	−0.05
**AIS 5 extremities > 1**	0.12‡	0.15‡	0.13‡	0.21‡	0.13‡	0.08†	0.07*	−0.01	−0.02
**AIS 5 extremities > 2**	0.06*	0.11‡	0.09†	0.19‡	0.10†	0.02	0.04	−0.06	0.00
**GOS < 5 (not well recovered)**	0.03	0.07*	0.07*	0.11‡	0.06*	0.09†	0.09†	0.12‡	0.15‡
**Hospital stay > 21 days**	0.17‡	0.17‡	0.19‡	0.27‡	0.21‡	0.10†	0.12‡	0.04	0.08†
**Not discharged home**	0.13‡	0.20‡	0.18‡	0.28‡	0.22‡	0.14‡	0.14‡	0.15‡	0.22‡
**Not discharged home or hospital stay > 21 days**	0.15‡	0.21‡	0.20‡	0.30‡	0.23‡	0.15‡	0.15‡	0.16‡	0.21‡

* *p* < 0.05; † *p* <0.01; ‡ *p* < 0.001. §Age at time of trauma of at least 80 years also showed lower or no significant correlations and are not shown. TOP, Trauma Outcome Profile; SF-36, Short Form-36; QOLIBRI, Quality Of Life after Brain Injury; ISS, Injury Severity Score; ICU, intensive care unit; GCS, Glasgow Coma Scale; AIS, Abbreviated Injury Scale (body regions 1–6); GOS, Glasgow Outcome Scale.

Correlations calculated separately for patients with and without TBI only showed minor differences, compared to the total correlation matrix with (*Tables S2–S4*). Comparing TBI with non-TBI patients, the latter group showed higher limitations for somatic aspects, whereas head injuries were more often followed by cognitive impairment, but differences were small (maximum *R*^2^ = 0.02).

In subsequent stepwise logistic regression analysis, the tested variables explained 22.4 to 22.8 per cent of the variance for somatically, 9.0 to 10.7 per cent for psychological, and 9.1 to 13.6 per cent for cognitively impaired outcome scores (*Table 3*).

**Table 3:** Stepwise logistic regression analysis of variables of somatically, psychologically, and cognitively impaired outcomes

	Nagelkerke *R*^2^			
	Per step	Total	Per step	Total
**Somatic outcome**	SF-36		TOP	
Functional restriction or pain before injury impaired (TOP)	0.063	0.063	0.152	0.152
AIS 5 extremities > 1	0.059	0.122	0.026	0.178
Age at time of trauma of at least 80 years	0.081	0.140	0.156	0.182
Age-unadjusted Charlson Comorbidity Index > 1	0.070	0.151	–	–
Not discharged home or hospital stay > 21 days	0.154	0.224	0.072	0.228
**Psychological outcome**	SF-36		TOP	
Functional restriction or pain before injury impaired (TOP)	0.040	0.040	0.052	0.052
No vocational education	0.018	0.058	0.024	0.076
ICU stay	0.051	0.069	0.066	0.090
AIS 5 extremities > 1	0.029	0.080	0.032	0.098
Not discharged home or hospital stay > 21 days	0.061	0.090	0.075	0.107
**Cognitive outcome**	QOLIBRI		TOP	
Functional restriction or pain before injury impaired (TOP)	0.043	0.043	0.063	0.063
AIS 1 head/neck > 2	0.018	0.061	0.026	0.089
No vocational education	0.053	0.071	0.070	0.096
Not discharged home or hospital stay > 21 days	0.038	0.091	0.066	0.136

SF-36, Short Form-36; TOP, Trauma Outcome Profile; AIS, Abbreviated Injury Scale (body regions 1–6); ICU, intensive care unit; QOLIBRI, Quality Of Life after Brain Injury.

**Fig. 2:**
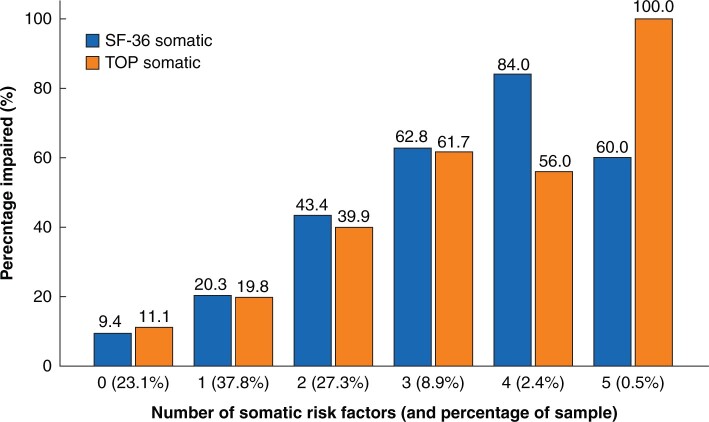
Somatic longer-term outcome: distribution of longer-term impaired outcome according to number of risk factors (preinjury pain or functional restriction, age at time of trauma of at least 80 years, age-unadjusted Charlson Comorbidity Index > 1, AIS 5 extremities > 1, not discharged home or hospital stay > 21 days) AIS, Abbreviated Injury Scale (body regions 1–6); SF-36, Short Form-36; TOP, Trauma Outcome Profile. Somatic subscores for SF-36 and TOP.

**Fig. 3:**
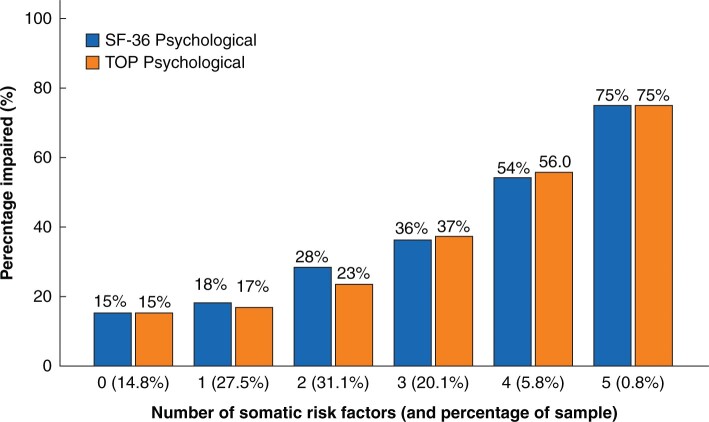
Psychological longer-term outcome: distribution of longer-term impaired outcome according to number of risk factors (preinjury pain or functional restriction, no vocational education, AIS 5 extremities > 1, ICU stay, not discharged home or hospital stay >21 days) AIS, Abbreviated Injury Scale (body regions 1–6); ICU, intensive care unit; SF-36, Short Form-36; TOP, Trauma Outcome Profile. Psychological subscores for SF-36 and TOP.

**Fig. 4:**
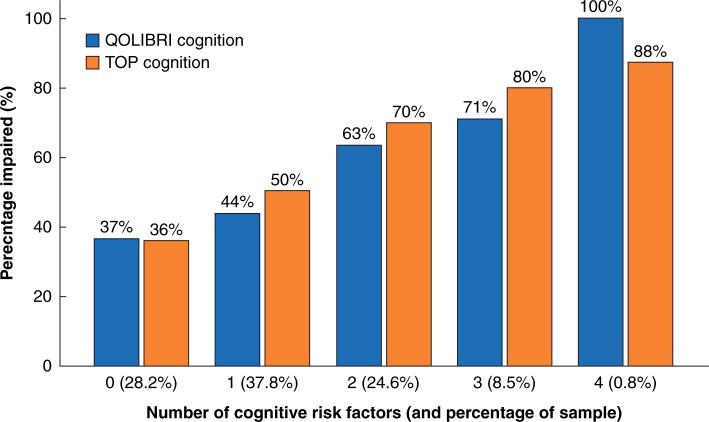
Cognitive longer-term outcome: distribution of longer-term impaired outcome according to number of risk factors (preinjury pain or functional restriction, no vocational education, AIS 1 head/neck > 2, not discharged home or hospital stay > 21 days) AIS, Abbreviated Injury Scale (body regions 1–6); QOLIBRI, Quality Of Life after Brain Injury; TOP, Trauma Outcome Profile. Cognitive subscores for QOLIBRI and TOP.

**Fig. 5:**
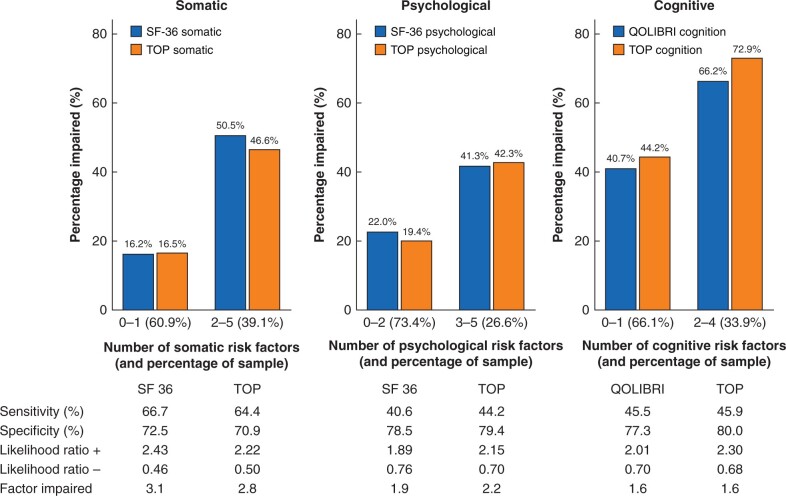
Sensitivity and specificity analysis of investigated scores indicating high-risk patients for specified reduced longer-term outcomes (ATPLOS procedure) SF-36, Short Form-36; TOP, Trauma Outcome Profile; QOLIBRI, Quality Of Life after Brain Injury; and specific subscores.

Based on this multivariate analysis, three prognostic scores (ATPLOS) were constructed from a total of eight risk factors: five for each of somatic and psychological impairment, and four for cognitive impairment. The criteria for each score are summarized in *Table 3*. *Table 4* translates these items into an assessment for clinical practice, including those patients not tested with the TOP questionnaire. The number of appropriate items is then calculated for each of the three dimensions. *Figs. 2–4* illustrate the distribution and percentage of longer-term impaired outcomes according to the resultant number of risk factors. More than two risk factors for somatic and cognitive impairment and more than three for psychological impairment showed a higher percentage of impaired patients than the total sample, and were therefore used as threshold values for the binary prediction scores, as shown in *Fig. 5*. Longer-term somatically impaired outcome was found to be a more reliable predictor than psychological or specific cognitive outcome. Consequently, 46.6 per cent (using the somatic subscore of the TOP) to 50.5 per cent (using the somatic subscore of the SF-36) of patients with longer-term somatically impaired outcomes were predicted with the newly developed risk score. Regarding observed sensitivity, specificity, and likelihood ratios (*Fig. 5*), the corresponding predictability was 2.8–3.1 times higher in high-risk patients than in those with a low-risk score (16.2 to 16.5 per cent). The predictability of risk scoring for longer-term psychological outcome (using psychological subscores of both SF-36 and TOP) was 1.9–2.2 times higher. For specific cognitive outcome (using cognitive factors of the QOLIBRI and TOP, respectively), predictability was consistently 1.6 times higher in high- *versus* low-risk patients.

**Table 4:** Translation of risk factors from the ATPLOS in everyday clinical practice

Item	Dimension*			Operationalization
	Som	Psy	Cog	
Functional restriction or pain before injury impaired (TOP)	x	x	x	The TOP asks about pain, functional limitations, and the suffering caused by them Patients who report severe pain or an objectively impairing functional limitation prior to the accident (for example, difficulty walking) may be considered to be at risk
Age at time of trauma of at least 80 years	x			Demography
No vocational education		x	x	No vocational education is often combined with the fact that patients do not have a secure and satisfying job
Age-unadjusted Charlson Comorbidity Index > 1	x			At least two of the following diagnoses: myocardial infarction, congestive heart failure, peripheral vascular disease, dementia, cerebrovascular disease, chronic lung disease, connective tissue disease, ulcer, chronic liver disease, diabetes AND/OR At least one of the following diagnoses: hemiplegia, moderate or severe kidney disease, diabetes with end-organ damage, tumour, leukaemia, lymphoma, moderate or severe liver disease, malignant tumour, metastasis, AIDS
AIS 5 extremities > 1	x	x		Abbreviated Injury Scale
AIS 1 head/neck > 2			x	Abbreviated Injury Scale
ICU stay		x		Patients who have to be treated in the ICU, mostly combined with artificial ventilation
Not discharged home or hospital stay > 21 days	x	x	x	Patients with long inpatient treatment (at least 3 weeks) or inpatient rehabilitation programme

*At least two items for the somatic and cognitive dimensions and at least three items for the psychological dimension should be present for an increased risk of long-time impairment. ATPLOS, Aarau trauma prognostic longer-term outcome scoring; Som, somatic; Psy, psychological; Cog, cognitive; TOP, Trauma Outcome Profile; AIDS, acquired immune deficiency syndrome; AIS, Abbreviated Injury Scale (body regions 1–6); ICU, intensive care unit.

## Discussion

This prospective study is the largest European trauma centre evaluation contemporaneously comparing general (SF-36) and trauma-specific (TOP, QOLIBRI) longer-term PROMs in non-selected patients following significant injury (NISS greater than or equal to 8). Briefly summarized, about every second injured individual reported cognitive limitations. Even though a range of socioeconomic, trauma, and treatment variables were investigated, correlations with longer-term outcome in this evaluation were, at best, weak, given their low explained variance. The elaborated prognostic scoring tool (ATPLOS) had a reasonable sensitivity and specificity to serve as an additional diagnostic instrument to detect impaired patients more easily.

In addition to the well known high rate of somatic and psychological restrictions noted by patients 1–2 years following trauma^10^^,^^32^^,^^37^^,^^38^, regardless of the measurement used, around half of injured patients reported cognitive limitations. The literature on longer-term outcomes after trauma reports varying rates of subsequent somatic, psychological, and cognitive impairment, depending on the trauma cohort analysed, as well as on patient differences regarding health status before injury, trauma severity, type of injuries, and treatment received^39^^,^^40^. Currently, data on psychological or even cognitive impairment following non-selected trauma (rather than head injury only, for example) are still rare^41–44^. As demonstrated in earlier work^22^, findings indicate that every second significantly injured person has residual cognitive impairment, almost independently of whether patients sustained brain injury or not. The possible therapeutic implications in terms of specific case management could make a great difference^41^ if vulnerable patients could be identified soon after trauma. Nevertheless, given the increasing interest in the psychological recovery and mental health of patients following physical injury^41^^,^^44^, future studies in mixed trauma cohorts are needed to confirm these pilot results. These studies may further substantiate the findings by relating the relevant PROM results to specific psychological diagnostics.

Although a range of socioeconomic, trauma, and treatment variables were investigated, correlations with longer-term outcome in this evaluation are, at best, weak, given their low explained variance. In line with other studies, univariate analysis was performed and found the highest correlations for preinjury pain or functional impairment (maximum *R* = 0.38). The fact that the majority of patients in the Brabant study^45^ sustained minor trauma (ISS < 8), with the authors reporting a far higher correlation, does indeed support the interpretation that such associations may decrease with the severity of the injury sustained. Surprising to any clinician is the result that trauma severity has a relatively low impact on longer-term outcome, as confirmed for musculoskeletal injuries (AIS 5) and somatic impairments (*R* = 0.13–0.21). This is equally valid for the low associations found in multivariate models, whereby the information available from all variables under investigation during hospital stay taken together explained a maximum of 23 per cent of the variance for somatic, and 11 to 14 per cent for psychological or cognitive (respectively) longer-term outcome. Comparison of the general and condition-specific PROMs used in this study found no important differences in the detection of patients’ longer-term deficits. This contrasts with the increasing consensus to use a combination of generic and condition-specific tools^1^^,^^9^^,^^19^ in investigations of trauma-specific sequelae. Given the finding that the TOP^22^ may discriminate cognitive deficits at least equally as well as the QOLIBRI in this context^22^, it is astonishing that no study outside Europe has used this PROM. Furthermore, neither the original US-American publications on the alternative trauma outcome scores Trauma-Qol (T-QoL) and RevisedTrauma-Qol (RT-QoL)^46^^,^^47^, which were developed later and lack cognitive dimensions, nor recent consensus conferences^9^ cite or comment on the previously developed TOP.

In daily work, clinicians lack instruments that allow prognostic identification of worse longer-term outcomes early in treatment of the traumatized patient, that is in time for further diagnostics and therapeutic interventions. Therefore, despite the low statistical associations observed, it was of interest to see how accurately a simple early treatment score, designed based on the results of the multivariate analysis, might identify patients at high risk of subsequent subjective deficits 1–2 years after injury. This pilot procedure resulted from the observation that most other investigations, although describing possible correlations^39^^,^^40^^,^^48^, did not offer any diagnostic cutoff values indicative of cases likely to have an unsatisfactory longer-term outcome. By applying specific limits, as published by the original authors of the PROMs, and used here combined with the significant factors found earlier in the logistic regression analysis, a prognostic scoring tool was constructed (ATPLOS). With this pilot scoring tool, it was possible to predict a longer-term somatically impaired outcome, with a sensitivity of 64 to 67 per cent and a specificity of 71 to 73 per cent, depending on the PROM used. The ATPLOS scoring achieved a sensitivity of 40 to 44 per cent and a specificity of 79 per cent for psychological outcome, and 46 per cent and 77 to 80 per cent, respectively, for cognitive outcome. Indisputably, from a statistical point of view, this predictive power is relatively weak in the context of prognostic testing. This may be related to the fact that multiple individual social and psychological factors, for example, may interfere with healing and non-healing^41^. As is known from other diseases, a plethora of elusive personal factors may interfere with individual outcome. For example, in terms of longer-term pain, frequently cited predictive factors include symptoms of anxiety and depression, patient perception that the injury was attributable to an external source (that is, they were not at fault), or cognitive avoidance of distressing thoughts^49^. Such individual factors may be captured by diagnostic interviews, but poorly by current systematic trauma assessments. Moreover, from a therapeutic point of view, an individual’s willingness to accept and integrate trauma was found to be imperative for healing^50^.

Despite these obvious limitations, and in the absence of well evidenced alternatives, ATPLOS can be used as an additional diagnostic instrument to identify impaired patients more easily. Depending on the pathology suspected, supplemental psychological or cognitive testing and support may be indicated, in addition to routine physical and occupational therapy approaches. Given the finding that 40 per cent of the patients in the study were diagnosed from their TOP scores with longer-term PTSD, a percentage that corresponds well with reports in the literature^44^, it can be inferred that early identification of high-risk individuals could facilitate early preventive therapy. Further studies are needed to confirm these pilot results.

The study findings must be interpreted in the light of the specific study cohort and the definitions used. The results are limited to the answers on the self-rated questionnaire returned by survivors of significant injury (defined as NISS greater than 8) who were all patients consecutively treated in a single Swiss trauma centre. It is not possible to comment on PROMs other than those investigated in this study. At first glance, a 43 per cent response rate 1 to 2 years after trauma may appear low. However, it is comparable with other reports on extended follow-up controls in the severely injured that included a bundle of standardized outcome instruments^20^^,^^51–55^; indeed, several larger studies have reported lower response rates^39^^,^^56^. Even short telephone interviews increasingly attract declining response rates, in comparison to earlier evaluations^40^. Furthermore, the characteristics of non-respondents differed little from those of respondents. The overall results of this study at a European trauma centre can be considered adequately representative of a consecutive cohort of significantly injured patients. This study was not primarily designed to construct or validate a new instrument to identify patients at high risk of poor longer-term outcome. To confirm these first results, the next step would be to examine patients neuropsychologically, both soon after injury and 1 to 2 years later. At present, it is not possible to comment on the possible impact of individual rehabilitation programmes being implemented following the application of the ATPLOS.

This study of significantly injured patients showed a high rate of somatic and psychological impairments, but particularly cognitive deficits. Despite the finding that early sociodemographic, trauma, and treatment variables correlated only to a limited extent with the outcome 1 to 2 years after trauma, multivariate analysis allowed the development of a potential diagnostic instrument, the ATPLOS, to predict the longer-term outcome of patients by using the PROMs SF-36 and TOP with reasonable sensitivity, specificity, and likelihood ratio. The application of such predictive factors may help clinicians to initiate well targeted therapeutic interventions earlier and more effectively.

## Supplementary Material

zrab109_Supplementary_Data
