# Supplementary material for: Main factors predicting somatic, psychological, and cognitive patient outcomes after significant injury: a pilot study of a simple prognostic tool
Source: BJS Open. 2021 Dec 3;5(6):zrab109. doi: 10.1093/bjsopen/zrab109 (PMC8643586; doi:10.1093/bjsopen/zrab109)
Supplement: zrab109_Supplementary_Data [file zrab109_supplementary_data.docx]

Table S1: Description and comparison of non-respondents and respondents

|  | Non-respondents (N=1399) | Respondents (N=1055) | Comparison | |
| --- | --- | --- | --- | --- |
|  | Mean (SD)  *Median (Interquartile)* | Mean (SD)  *Median (Interquartile)* | R^2^ | P |
| Age at time of trauma (years) | 55.5 (22.0) | 54.1 (19.2) | 0.00 | .096 |
| ISS | 13.6 (7.1)  *11 (9-17)* | 14.0 (7.6)  *12 (9-17)* | 0.00 | .227 |
| NISS | 18.0 (9.1)  *17 (11-22)* | 18.6 (9.5)  *17 (12-22)* | 0.00 | .098 |
| RISC (%) | 8.1 (12.2)  *3.0 (1.1-9.3)* | 7.1 (11)  *3.0 (1.1-8.8)* | 0.00 | .037 |
| AIS1 head/neck | 1.91 (1.56)  *2 (0-3)* | 1.70 (1.56)  *2 (0-3)* | 0.00 | .001 |
| AIS2 face | 0.34 (0.76)  *0 (0-0)* | 0.40 (0.83)  *0 (0-0)* | 0.00 | .083 |
| AIS3 chest | 0.95 (1.38)  *0 (0-2)* | 1.09 (1.44)  *0 (0-3)* | 0.00 | .015 |
| AIS4 abdomen | 0.43 (0.95)  *0 (0-0)* | 0.50 (1.03)  *0 (0-0)* | 0.00 | .059 |
| AIS5 extremities | 1.09 (1.25)  *0 (0-2)* | 1.22 (1.28)  *0 (1-2)* | 0.00 | .010 |
| AIS6 external | 0.42 (0.57)  *0 (0-1)* | 0.42 (0.56)  *0 (0-1)* | 0.00 | .807 |
| Hospitalisation (days) | 9.7 (9.1)  *7 (4-13)* | 12.1 (10.9)  *10 (5-16)* | 0.01 | .000 |
|  | n (%) | n (%) | R^2^ | P |
| Age at time of trauma at least 80y * | 260 (18.6%) | 92 (8.7%) | 0.02 | .000 |
| Gender, female | 430 (30.7%) | 356 (33.7%) | 0.00 | .114 |
| Living in a partnership * | - | 568 (53.8%) | - | - |
| No vocational education * | - | 174 (16.5%) | - | - |
| TOP: Preinjury function or pain impairment ** | - | 164 (15.5%) | - | - |
| Age unadjusted Charlson Comorbidity Index >1 | 192 (13.7%) | 95 (9.0%) | 0.01 | .000 |
| High trauma energy | 588 (42.5%) | 567 (54.1%) | 0.01 | .000 |
| Traffic collision except car | 444 (32.1%) | 426 (40.6%) | 0.01 | .000 |
| ISS ≥16 | 447 (32.0%) | 371 (35.2%) | 0.00 | .095 |
| ICU stay | 552 (39.5%) | 497 (47.1%) | 0.01 | .000 |
| Intubation | 216 (15.4%) | 212 (20.1%) | 0.00 | .003 |
| 1^st^ GCS <13 | 155 (11.1%) | 148 (14.0%) | 0.00 | .028 |
| AIS1 head /neck >1 | 838 (59.9%) | 580 (55.0%) | 0.00 | .014 |
| AIS1 head /neck >2 | 616 (44.0%) | 368 (34.9%) | 0.01 | .000 |
| AIS 3 chest >1 | 460 (32.9%) | 395 (37.4%) | 0.00 | .019 |
| AIS3 chest >2 | 325 (23.2%) | 282 (26.7%) | 0.00 | .047 |
| AIS4 abdomen >1 | 253 (18.1%) | 218 (20.7%) | 0.00 | .108 |
| AIS4 abdomen >2 | 67 (4.8%) | 70 (6.6%) | 0.00 | .049 |
| AIS5 extremities >1 | 616 (44.0%) | 516 (48.9%) | 0.00 | .016 |
| AIS5 extremities >2 | 232 (16.6%) | 214 (20.3%) | 0.00 | .019 |
| GOS <5 (not well recovered) | 326 (23.3%) | 217 (20.9%) | 0.00 | .164 |
| Hospital stay >21d | 133 (9.5%) | 136 (12.9%) | 0.00 | .008 |
| Not discharged home | 673 (48.1%) | 490 (46.5%) | 0.00 | .415 |
| Not discharged home or hospital stay >21d | 690 (49.3%) | 515 (48.8%) | 0.00 | .804 |

* Age at least 70 or age at least 60 did not correlate with outcome values and are therefore not shown

** collected for respondents only;

SD, Standard Deviation; R^2^, explained variance, (N)ISS, (New) Injury Severity Score; RISC, Revised Injury Severity Classification (%); AIS, Abbreviated Injury Scale (body regions 1-3); TOP, Trauma Outcome Profile; ICU, Intensive Care Unit; GCS, Glasgow Coma Score; GOS, Glasgow Outcome Scale.

Table S2: Univariate correlation of socio-demographic, injury, treatment, and hospital-outcome variables with somatic, psychological, and cognitive impaired longer-term outcome for patients without TBI (AIS1<2; N=475)

|  | TOP: Pain impairment | TOP: Function restricted | TOP: Function or pain impairment | SF-36: Somatic impairment | TOP: Somatic impairment | SF-36 Psychological impairment | TOP Psychological impairment | QOLIBRI: Cognition impairment | TOP: Cognitive impairment |
| --- | --- | --- | --- | --- | --- | --- | --- | --- | --- |
| Age at time of trauma at least 80y | 0.06 | 0.10* | 0.09 | 0.21*** | 0.15*** | -0.03 | -0.02 | 0.09 | 0.20*** |
| Gender, female | 0.01 | 0.05 | 0.04 | 0.05 | 0.07 | 0.08 | 0.04 | -0.05 | 0.06 |
| Living in a partnership | 0.00 | -0.02 | -0.01 | -0.04 | -0.04 | -0.09* | -0.03 | -0.01 | 0.04 |
| No vocational education | 0.14** | 0.09* | 0.15*** | 0.04 | 0.13** | 0.09* | 0.13** | 0.09* | 0.07 |
| Age unadjusted Charlson Comorbidity Index >1 | 0.05 | 0.06 | 0.05 | 0.16*** | 0.05 | 0.06 | 0.03 | 0.05 | 0.11* |
| TOP: Preinjury function or pain impairment before trauma | 0.35*** | 0.39*** | 0.35*** | 0.27*** | 0.39*** | 0.18*** | 0.20*** | 0.23*** | 0.25*** |
| High trauma energy | 0.01 | -0.05 | 0.00 | -0.05 | -0.03 | -0.01 | 0.00 | -0.04 | -0.04 |
| Traffic collision except car | -0.01 | -0.04 | -0.02 | -0.10* | -0.04 | 0.02 | 0.00 | 0.00 | -0.04 |
| ISS ≥16 | 0.00 | 0.02 | 0.05 | -0.02 | 0.01 | 0.00 | -0.03 | 0.01 | 0.05 |
| ICU stay | 0.01 | 0.08 | 0.05 | 0.04 | 0.07 | 0.13** | 0.10* | 0.01 | 0.10* |
| Intubation | 0.03 | 0.02 | 0.04 | 0.03 | 0.05 | 0.04 | 0.14** | 0.03 | 0.07 |
| 1st GCS <13 | 0.07 | 0.06 | 0.05 | 0.03 | 0.07 | 0.02 | 0.04 | 0.02 | 0.06 |
| AIS3 chest >1 | -0.10* | -0.07 | -0.08 | -0.15*** | -0.10* | -0.10* | -0.06 | -0.01 | -0.06 |
| AIS3 chest >2 | -0.15** | -0.07 | -0.11* | -0.15*** | -0.12* | -0.08 | -0.06 | 0.01 | -0.06 |
| AIS4 abdomen >1 | 0.04 | 0.02 | 0.07 | -0.02 | 0.01 | -0.05 | -0.01 | -0.05 | 0.02 |
| AIS4 abdomen >2 | -0.04 | -0.07 | -0.02 | -0.06 | -0.04 | -0.02 | -0.01 | -0.09* | -0.04 |
| AIS5 extremities >1 | 0.09* | 0.13** | 0.11* | 0.16*** | 0.10* | 0.12** | 0.12** | 0.02 | 0.07 |
| AIS5 extremities >2 | 0.02 | 0.08 | 0.06 | 0.16*** | 0.08 | -0.01 | 0.03 | -0.03 | 0.08 |
| GOS <5 (not well recovered) | 0.13** | 0.12** | 0.13** | 0.10* | 0.12* | 0.09* | 0.11* | 0.04 | 0.08 |
| Hospital stay >21d | 0.15*** | 0.15*** | 0.16*** | 0.19*** | 0.18*** | 0.10* | 0.13** | 0.00 | 0.07 |
| Not discharged home | 0.16*** | 0.17*** | 0.18*** | 0.28*** | 0.23*** | 0.12** | 0.12** | 0.08 | 0.17*** |
| Not discharged home or hospital stay >21d | 0.18*** | 0.18*** | 0.21*** | 0.31*** | 0.25*** | 0.13** | 0.14** | 0.1* | 0.18*** |

SF-36, The Short Form-36; TOP, Trauma Outcome Profile; QOLIBRI, the Quality Of Life after Brain Injury score; (N)ISS, (New) Injury Severity Score; RISC, Revised Injury Severity Classification (%); AIS, Abbreviated Injury Scale (body regions 1-6); ICU, Intensive Care Unit; GCS, Glasgow Coma Score; GOS, Glasgow Outcome Scale.

Table S3: Univariate correlation of socio-demographic, injury, treatment, and hospital-outcome variables with somatic, psychological, and cognitive impaired longer-term outcome for patients with TBI (AIS1>1; N=580)

|  | TOP: Pain impairment | TOP: Function restricted | TOP: Function or pain impairment | SF-36: Somatic impairment | TOP: Somatic impairment | SF-36 Psychological impairment | TOP Psychological impairment | QOLIBRI: Cognition impairment | TOP: Cognitive impairment |
| --- | --- | --- | --- | --- | --- | --- | --- | --- | --- |
| Age at time of trauma at least 80y | 0.03 | 0.07 | 0.05 | 0.09* | 0.07 | 0.02 | 0.01 | 0.08* | 0.11** |
| Gender, female | 0.02 | 0.06 | 0.05 | -0.04 | 0.03 | 0.07 | 0.01 | 0.01 | 0.08* |
| Living in a partnership | 0.02 | 0.04 | 0.04 | 0.03 | 0.04 | 0.03 | -0.03 | -0.04 | 0.07 |
| No vocational education | 0.13*** | 0.05 | 0.08 | 0.08* | 0.11** | 0.15*** | 0.15*** | 0.11** | 0.11** |
| Age unadjusted Charlson Comorbidity Index >1 | 0.1* | 0.12** | 0.15*** | 0.13** | 0.13** | 0.04 | 0.02 | 0.06 | 0.05 |
| TOP: Preinjury function or pain impairment before trauma | 0.35*** | 0.33*** | 0.36*** | 0.18*** | 0.32*** | 0.17*** | 0.2*** | 0.14*** | 0.19*** |
| High trauma energy | -0.02 | -0.03 | -0.02 | 0.09* | -0.04 | -0.04 | -0.02 | -0.03 | -0.05 |
| Traffic collision except car | -0.06 | -0.03 | -0.03 | 0.11* | -0.06 | -0.01 | -0.01 | -0.01 | -0.02 |
| ISS ≥16 | -0.02 | 0.07 | 0.03 | 0.12** | 0.05 | 0.02 | 0.09* | 0.01 | 0.09* |
| ICU stay | -0.01 | 0.05 | 0.03 | 0.06 | 0.02 | 0.06 | 0.08* | 0.09* | 0.12** |
| Intubation | -0.02 | 0.1* | 0.05 | 0.13** | 0.09* | 0.03 | 0.01 | 0.03 | 0.08 |
| 1st GCS <13 | -0.06 | 0.03 | -0.01 | 0.06 | 0.03 | 0 | 0.01 | 0.04 | 0.09* |
| AIS3 chest >1 | 0.1* | 0.11** | 0.09* | 0.19*** | 0.09* | 0 | 0.06 | -0.03 | 0.04 |
| AIS3 chest >2 | 0.08* | 0.1* | 0.07 | 0.16*** | 0.07 | -0.01 | 0.02 | -0.05 | 0 |
| AIS4 abdomen >1 | 0.04 | 0.04 | 0.04 | 0.05 | 0.03 | 0.02 | 0.03 | -0.03 | 0.03 |
| AIS4 abdomen >2 | 0.01 | -0.02 | -0.01 | 0.04 | -0.02 | -0.03 | 0 | -0.02 | -0.01 |
| AIS5 extremities >1 | 0.1* | 0.13** | 0.1* | 0.18*** | 0.12** | 0.07 | 0.07 | 0 | -0.01 |
| AIS5 extremities >2 | 0.05 | 0.1* | 0.07 | 0.15*** | 0.09* | 0.07 | 0.09* | -0.06 | -0.01 |
| GOS <5 (not well recovered) | -0.01 | 0.05 | 0.05 | 0.15*** | 0.04 | 0.08 | 0.07 | 0.16*** | 0.18*** |
| Hospital stay >21d | 0.17*** | 0.19*** | 0.21*** | 0.33*** | 0.22*** | 0.1* | 0.12** | 0.09* | 0.12** |
| Not discharged home | 0.12** | 0.24*** | 0.19*** | 0.29*** | 0.22*** | 0.16*** | 0.16*** | 0.21*** | 0.25*** |
| Not discharged home or hospital stay >21d | 0.12** | 0.24*** | 0.2*** | 0.3*** | 0.22*** | 0.16*** | 0.15*** | 0.21*** | 0.24*** |

SF-36, The Short Form-36; TOP, Trauma Outcome Profile; QOLIBRI, the Quality Of Life after Brain Injury score; (N)ISS, (New) Injury Severity Score; RISC, Revised Injury Severity Classification (%); AIS, Abbreviated Injury Scale (body regions 1-6); ICU, Intensive Care Unit; GCS, Glasgow Coma Score; GOS, Glasgow Outcome Scale.

Table S4: Comparison of somatic, psychological, and cognitive impaired longer-term outcomes for patients with and without TBI (AIS 1 head/neck >1)

|  | Total (N=1055) | Non-TBI (N=475) | TBI (N=580) |  |  |
| --- | --- | --- | --- | --- | --- |
|  | n (%) | n (%) | n (%) | R^2^ | P |
| TOP: Pain impairment | 580 (28.1%) | 159 (33.5%) | 141 (24.3%) | 0.01 | .001 |
| TOP: Function restricted | 276 (26.2%) | 144 (30.3%) | 134 (23.1%) | 0.01 | .008 |
| TOP: Function or pain impairment | 349 (33.1%) | 182 (38.3%) | 168 (29%) | 0.01 | .001 |
| SF-36: Somatic impairment | 310 (29.4%) | 173 (36.4%) | 139 (24%) | 0.02 | .000 |
| TOP: Somatic impairment | 297 (28.2%) | 152 (32%) | 146 (25.2%) | 0.01 | .014 |
| SF-36 Psychological impairment | 286 (27.1%) | 125 (26.3%) | 161 (27.8%) | 0.00 | .600 |
| TOP Psychological impairment | 271 (25.7%) | 109 (23%) | 160 (27.6%) | 0.00 | .086 |
| QOLIBRI: Cognition impairment | 521 (49.4%) | 218 (45.9%) | 303 (52.2%) | 0.00 | .040 |
| TOP: Cognitive impairment | 570 (54%) | 226 (47.6%) | 343 (59.1%) | 0.01 | .000 |

TBI, Traumatic Brain Injury; SF-36, The Short Form-36; TOP, Trauma Outcome Profile; QOLIBRI, the Quality Of Life after Brain Injury score.
